# A Driving Behaviour Model of Electrical Wheelchair Users

**DOI:** 10.1155/2016/7189267

**Published:** 2016-04-11

**Authors:** S. O. Onyango, Y. Hamam, K. Djouani, B. Daachi, N. Steyn

**Affiliations:** ^1^F'SATI, Tshwane University of Technology, Private Bag Box X680, Staatsartillerie Road, Pretoria West, Pretoria 0001, South Africa; ^2^Laboratoire d'Informatique Avancée de Saint de Denis (LIASD), University of Paris 8, France

## Abstract

In spite of the presence of powered wheelchairs, some of the users still experience steering challenges and manoeuvring difficulties that limit their capacity of navigating effectively. For such users, steering support and assistive systems may be very necessary. To appreciate the assistance, there is need that the assistive control is adaptable to the user's steering behaviour. This paper contributes to wheelchair steering improvement by modelling the steering behaviour of powered wheelchair users, for integration into the control system. More precisely, the modelling is based on the improved Directed Potential Field (DPF) method for trajectory planning. The method has facilitated the formulation of a simple behaviour model that is also linear in parameters. To obtain the steering data for parameter identification, seven individuals participated in driving the wheelchair in different virtual worlds on the augmented platform. The obtained data facilitated the estimation of user parameters, using the ordinary least square method, with satisfactory regression analysis results.

## 1. Introduction

*(1) Motivation.* The manoeuvring difficulty experienced by wheelchair users with Parkinson's disease, multiple sclerosis, and related handicaps is the main motivation for this study. Such handicaps complicate the ability to effectively manipulate the conventional joystick, even within fairly simple environments [1]. According to Fehr et al. [2], about 40% of the users struggle to steer the standard powered wheelchair with ordinary user interfaces. Fehr et al. observe that close to 50% of the affected user group can be assisted if better control methods, with supplemented user interfaces and/or support systems capable of accommodating their needs and preferences, were employed. Huge research on joysticks and related interfaces including haptic systems has emerged [3–7], and new control models [8, 9] are continuing to develop. The available driver models however suffer lack of individuality [10], focusing mostly on the common user attributes, and assume that all users respond to particular navigational situations by similar general patterns. Such driver models employ general parameters that barely correspond to measurements obtained from extreme users and hardly take into consideration the contextual nature of human response to stimuli. Besides, the available control and assistive techniques rarely consider the fact that the steering capability of users with degenerative conditions, like ageing, deteriorates progressively over time. Adapting the wheelchair to the user's best steering behaviour may simplify the general steering task and limit the steering troubles attributable to the worsening disability condition of the user. This necessitates modelling and a priori identification of the driver's steering behaviour.

*(2) Background.* Although extensive information regarding modelling and control of powered wheelchairs exists [11–17], behaviour modelling for assistance and rehabilitation is still limited. In fact, apart from [18–23], the authors failed to locate more behaviour models related to wheelchair drivers. Studies in the vast area of behaviour modelling have been approached mainly from the field of automotives, aviation simulators, and robotic intelligence [24, 25]. The existing formulations in these areas have been modified progressively from linear to empirical models, with the hope of finding general models that represent the operators' behaviour [26–28]. According to [29–32], most of the available driver models are qualitative, with elaborate explanations of the drivers' perception and vehicle handling techniques, while others entail detailed reports of the drivers' actions during normal driving situations. The common quantitative models available in the literature represent specific driving tasks [33–36]. The adaptive and comprehensive control models of the driving behaviour are, however, very few [37, 38].

According to Michon [39], the entire driving task has three demand levels, consisting of the strategic level, the tactical level, and the control level. Each level encompasses the driver, the wheelchair, and the environment. The three elements interact continuously, and every state of the vehicle can be linked to this interaction [18]. The driving tasks like risk avoidance and steering velocity determination are therefore entirely dependent upon the three elements. However, the adaptive behaviours executed within the driving environment are greatly controlled by preferences of the driver, which makes the driver a very important element in the driving task.

In the literature, hierarchical structures of car driving models have been presented in terms of skill based, rule based, and knowledge based behaviours [40]. However, it is hard to verify the contributions of such qualitative models of perceptual processes and neural activities, in the actual execution of the driving task. Pilutti and Galip Ulsoy [41] therefore considered the system identification approach using the back-box model with autoregressive exogenous structure (ARX) to identify the driver model's parameters. Chen and Ulsoy [42] also presented the same formulation approach for both driver model and model uncertainties, using the actual driving data captured from a fixed-base driving simulator. The model however employed the autoregression moving average with exogenous inputs (ARMAX) to improve on accuracy, based on the consideration that ARMAX can yield residuals closer to white noise with fewer parameters given the same model order. The system identification approach is also considered in [10]; nevertheless the authors do not take into account exogenous inputs in their affine autoregressive system, but instead derive a multistep model output error criterion and present an algorithm to identify the parameters of the subsystem using measurable motion data. Although the consideration of black-box method of system identification is straight forward and common with availability of data, authors believe that sufficient information can be found to relate the driver's actions to the perceivable contextual environment.

The study contributes to wheelchair driver behaviour modelling by formulating a simple steering model that is also linear in parameters. The complexity of the steering model is very instrumental in determining whether the model is applicable on-line, for real-time adaptation, or off-line, for periodic or permanent adaptation. Derivation of the presented model is based on deductive reasoning from the known steering operations and systematic relationships between the observable behaviours, taking into consideration the environmental situation. It however shuns the consideration of social events occurring within the driver's mind. In order to capture the adaptable demands of the driver at the control and tactical levels, the driver-specific parameters are identified. The steering data obtained from the augmented virtual-reality wheelchair platform, known as Virtual-Space 1 (VS-1) at FSATI (FSATI is an acronym for French South-African Institute of Technology) in TUT (TUT is an acronym for Tshwane University of Technology.) [43], is utilised in the identification of the model parameters. The identified parameters are then used to curve-fit and compare the model against the observed data.

**Figure 1:**
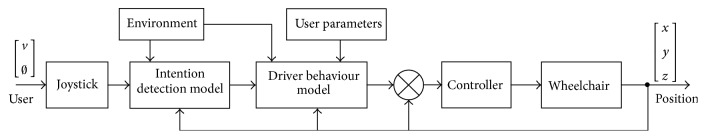
The control diagram of a wheelchair with integrated driver behaviour model and intention detection model.

The presented steering model can be used to adapt the wheelchair to the user's steering behaviour according to Figure 1. Due to its simplicity and linearity, the proposed model is applicable to wheelchair self-tuning adaptive control, to observe the preceding behaviour and self-tune the parameters to fit the observation.

This paper is organised as follows.  Section 1 has presented the introduction in terms of the motivation and background of behaviour modelling, taking into consideration the interactive elements involved in the accomplishment of steering tasks.  Section 2 presents some of the approaches that have previously been considered in the derivation of specific driver control models including the available wheelchair driver behaviour models. In Section 3, simulator evaluation and summary of the experiments conducted to obtain the necessary steering data for driver identification are presented. The driver behaviour model is presented in Section 4, while the statistical analysis of the model and its comparison to observed data are discussed in Section 5. Finally, conclusions and future recommendation are presented in Sections 6 and 7, respectively.

## 2. Related Path Planning Models and Driver Adaptation Literature

Driving begins when an optimal path to the destination is conceived. This involves careful consideration of the entire workspace. Based on the associated constraints, the conception is accomplished either fully in advance before setting out the journey or in parts within conceptually subdivided sections of the workspace, during the driving process. Path planning for robotic automation has been achieved by deliberate and reactive planners. Deliberate planners including cell decomposition, road-maps, and evolutionary algorithms ensure prior plan of the whole journey. However, they entail expensive computations that limit their practical application in higher dimensional configurations. Deliberate planners are, therefore, commonly applied to unmanned ground vehicle in confined environments. On the contrary, local planners provide cheap trajectory planning algorithms, based on sensor information captured from the local surrounding. Local planners ensure both faster and real-time update of the environmental information, as well as reactive response to stimuli. As a result, they are commonly aimed at ensuring safety and stability of both the driver and the vehicle. Nonetheless, the paths obtained from these approaches may not be optimal, and the vehicle could be trapped into local minima. This makes the application of local planners to unmanned mobile systems inefficient without deliberate planners. Both deliberate and reactive planners still command significant influence in the literature. However, the current focus is aimed mainly at integrating deliberate and reactive planners into unified structures, to overcome the drawback of individual planners. This explains the current increase in hybrid planners [44].

Although wheelchair steering also involves both deliberate and local planning, the actual control or steering behaviour of the users can be considered local. This is characterised by reactive adaptations that the user performs in response to perceived risks and undesired situations. Local planners can, therefore, be considered in the formulation of wheelchair drivers' steering behaviour. Besides, the driver's presence eliminates the common limitations of local planners, as he/she is personally available to solve the nonoptimality and the global (local minima and trap situations) problems. The common local planners in literature include nearness diagram, dynamic window, velocity obstacle, and potential field methods. The strength of nearness diagrams is based on the situation analysis performed by the system to select the new direction of motion that reduces the local minima. Both dynamic window and velocity obstacle approaches operate in the system's velocity space, by admitting all velocities that allow stopping without collision. However, they are computationally intensive, and only up to 1.0 m/s has been achieved with dynamic window approach according to [45]. The velocity obstacle approach also requires complete knowledge of other agents in the environment, including their future dynamics. Besides, the implementation of their analytical solutions is more difficult with environmental uncertainties and noisy data from the agents [46]. On the other hand, the potential field method is known to be “elegant” and compatible to most real-time problem solving tools with minimal computational demands.

### 2.1. The Potential Field Method

The Artificial Potential Field (APF) method is, therefore, considered in this study. The APF methods allocate the potential function in (1) in the configuration space, by representing the goal as an attractor and obstacles as repellers. The potential field function denoted as **U**_art_ is defined as sum of the attractive potential **U**_att_ and repulsive potential **U**_rep_. The force function can be obtained by computing the negative integral of **U**_art_:

(1) $$\begin{array}{c} U_{art}=U_{att}+U_{rep}. \end{array}$$

#### 2.1.1. Attractive Potential

The attractive force is, commonly, simply represented to attain its minimum at the intended goal [47–49]. However, because the driver is always available, in wheelchair steering, to provide the motivating force to the goal, the conventional Khatib's [47] attractive potential may be unnecessary in the wheelchair steering behaviour model.

#### 2.1.2. Repulsive Potential

The repulsive potential is often considered to have an inverse relationship with the square of obstacles distance *ρ*_obst_^2^. In the literature, the following representations are commonly used to express the repulsive potential.

*Minimum Distance Representation.* Here, the repulsive force is computed out of the minimum distance between the obstacle and the vehicle at time instant *k*:

(2) $$\begin{array}{c} F_{rep_{k}}=k_{o}\frac{1}{\min\left(\rho_{obst_{k}}^{2}\right)}, \end{array}$$

where *k*_*o*_ is a positive constant that scales the repulsive potential, while *ρ*_obst_(**q**, **q**_obst_) is the distance between the vehicle at position **q** and the obstacle at position **q**_obst_.

*Multiple Distance Representation*. Here, several *i* equidistant points on the obstacle are selected, and the repulsive force directed to the vehicle is computed at every time instant *k*, according to the following expression:

(3) $$\begin{array}{c} F_{rep_{k}}=\overset{N}{\underset{i=1}{\sum }}k_{o}\frac{1}{\rho_{obst_{i_{k}}}^{2}}. \end{array}$$

*Representation with Restricted Radius of Influence.* Latombe [50] proposed an adjustment to the conventional repulsive potential by limiting the radius of influence *ρ*_0_obst__ of the obstacle. This eliminates unnecessary obstacle effects on the vehicle when *ρ*_obst_*i*__ is large enough to allow safe passage:

(4) $$\begin{array}{c} U_{rep}=\left\{\begin{array}{cc} \frac{1}{2}k_{o}\left(\frac{1}{\rho_{obst}}-\frac{1}{\rho_{0_{obst}}}\right) & \rho_{obst}\leq \rho_{0_{obst}}\\ 0 & \rho_{obst}>\rho_{0_{obst}.} \end{array}\right. \end{array}$$

*Directed Potential Field (DPF).* The approach proposed by Taychouri et al. [51] is of special interest. Apart from using distance representation and taking into account the position of the obstacle and its direction of motion, it also allocates maximal repulsive potential whenever the vehicle is moving directly towards the obstacle and negligible potential whenever the obstacle is at right angle to the direction of motion. Due to its strength and ingenious simplicity, this formulation is considered to represent the subjective risk function of the driver's behaviour during steering:

(5) $$\begin{array}{c} F_{rep_{k}}=\overset{N}{\underset{i}{\sum }}k_{o}\frac{{\left(\cos\alpha_{i}\right)}^{m}}{\rho_{obst_{i_{k}}}^{2}}. \end{array}$$

In (5), *α*_*i*_ is the angle between point *i* of the obstacle and the direction of motion of the vehicle, while *m* is a gain constant.

The main causes of discontents that have seen several modifications in the potential field approach [52, 53] include the availability of local minima and trap situations [54], the nonoptimality problem, and the goals nonreachable with obstacles nearby (GNRON). Some of the recent APF modifications that have been proposed include the Evolutionary Artificial Potential Field (EAPF) method [55], which integrates the APF method with genetic algorithms, to derive an optimal potential field function that ensures global planning without local minima. The EAPF model uses both Multiobjective Evolutionary Algorithm (MOEA) to identify the most optimal potential field function and the escape force algorithm to avoid the local minima. In [53], the concept of Parallel Evolutionary Artificial Potential Field (PEAPF) is introduced as a new path planning method in mobile robot navigation. PEAPF improves the earlier EAPF method by making the controllability of the vehicle in real-world scenarios with dynamic obstacles possible. The recent Bacterial Evolutionary Algorithm (BEA) [56] also compares closely with PEAPF, introducing an enhanced flexible planner to improve the EAPF method. While these always solve the APF drawbacks, the logical consideration of these modifications with regard to real-time applications, in most cases, turns out to be unrealistic, because the resulting models involve expensive computational steps [57].

The important considerations in the approach of choice, for wheelchair drivers, include the features that enhance their adaptational behaviours within the local environment. This is because, unlike in robotics, the driver is always available in wheelchair steering to solve the globality problems. The authors therefore believe that global planning at the expense of computational simplicity may constitute a worthless trade-off especially for real-time applications. The features of consideration regarded in this study include computational complexity, path smoothness, context scalability, directionality, and handling capability in complex environments.

*Computational Complexity*. The implementation of a control model in a real-time application is strongly influenced by the amount time it takes to compute the control signal that generates both feasible path and desired speed. According to [57] it is more suitable to have a very fast path planner for real-time applications than to perceive a vehicle that only learns its workspace to memorise a variety of standard paths. A finite control behaviour encompassing only the perceivable workspace can be considered to increase planner's computational speed.

*Path Smoothness.* This regards the capability of the planner to interpret the dynamic and static behaviours of other agents within the workspace in order to execute the adaptive control without jerks. The response speed of the planner and the computed magnitude of the steering signal are very crucial in determining the quality of the resulting path. A driver model with smooth planning capability could be instrumental in the assistance of wheelchair users with disabilities like hand tremors and cognitive disorders.

*Context Scalability*. It is important that only behaviours of the agents that influence the driver's subjective risk are taken into consideration. Scaling down the entire workspace, for instance, to the area enclosed by the look-ahead radius and further to a smaller area encompassing the driver's field of view, may reduce the complexity of analysis and enhance the quality of control.

*Directivity*. This concerns the amount of influence imposed on the driver by virtue of the agent's position and the direction of motion of the vehicle. Directed models enhance smoother variations in the sensor signals and therefore influence the quality of the generated path.

*Handling Capability in Complex Environments*. The handling capability regards the computational speed of the planner and its ability to take into consideration the dynamic behaviour of other agents in the configuration space. 

Table 1 compares the features of some recent potential field methods with the proposed DPF.

### 2.2. The Available Wheelchair Driver Models in Literature

Most of the wheelchair driver models in the literature are concerned with detection of the user's intention in terms of the direction of travel rather than adaptation of the wheelchair to the user's steering behaviour. In [58, 59], for instance, an intelligent decision making agent is presented for driver's intention detection in uncertain local environments based on the Partially Observable Markov Decision Process (POMDP). Using the same methodology, a global intention recognition model is also presented in [60] for autonomous wheelchair navigation. Besides, a multihypothesis approach is considered in [61, 62] to predict the driver's intention and provide collaborative control, by adjusting the steering signal to avoid observable risks during navigation. The use of Bayesian networks for user intention recognition and estimation of uncertainty on the user's intent has also been considered [19, 20, 63, 64]. These Bayesian network approaches formulate the intended direction of the user on-line during navigation. Although the uncertainty involved is taken into consideration, such models do not incorporate the adaptable demand of the driver into the wheelchair.

Apart from the intention detection models, a filtering approach that presumes an experienced reference driver to eliminate the user handicap is considered in [22], while the task oriented models that generate autonomous behaviours at different levels are proposed in [65–70]. The task oriented models may allocate the driver more or less control depending on the contextual need at one level and enable the wheelchair to perform autonomous tasks without the driver's input at another level. In both cases, however, the resulting behaviour may not represent the actual steering preference of the user.

The reactive model of wheelchair driver behaviour that can be used to adapt wheelchair steering to the user's behaviour is proposed by [18]. The model is derived in terms of two force components: the driving force **F**_*d*_ (6) and the environmental or obstacle force **F**_*k*_ (7):

(6) Fd=KτVmax1−XsafXacte−Vact,

(7) Fk=koexp⁡−ρobstBnDv,

where *K* is the weight constant, *τ* is the driver's relaxation or reaction time, *V*_max_ is the maximum limit of wheelchair velocity, *X*_saf_ is the safe distance, **e** is a unit vector in the direction of motion, and *X*_act_ is the current wheelchair position. Additionally, *B* is a constant that represents the range of the repulsive force, **n** is a unit vector in the direction of the moving obstacle, and *D*_*v*_ is the directivity factor.

Although this model is good, it is nonlinear and does not represent in a simple way the diminishing influence of the risks positioned behind or perpendicular to the direction of the wheelchair's motion. In addition, it is not tested to real data.

## 3. Experiments and Acquisition of Steering Data

### 3.1. Evaluation of the VS-1 Simulator

**Figure 2:**
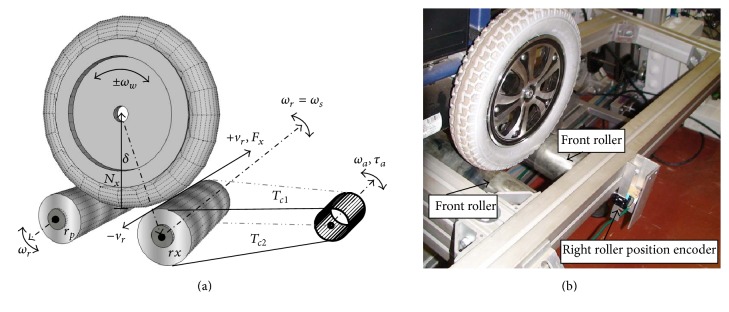
The roller system on the motion platform that enables both rotational motion of the wheels and the mapping of the wheel's motion into the virtual world.

**Figure 3:**
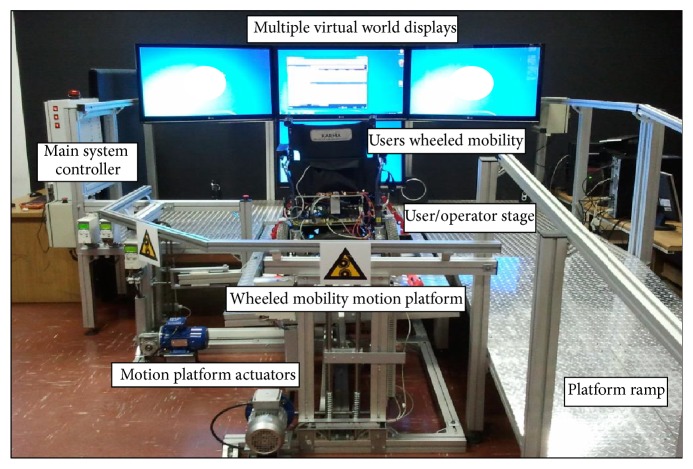
Virtual-Reality System 1 (VS-1): the augmented virtual and motion simulator at FSATI for wheelchair simulations.

The experiments to obtain the required steering data have been conducted on the VS-1 simulator [71] depicted in Figure 3. The platform's basic components include the visual interface, the motion platform, and the controller. The virtual interface presents to the user the synchronised virtual world either through stereoscopic Head Mounted Display (HMD) or through the four-connected screens display. The motion platform that consists of a user ramp and a stage can either host an electrical or a manual wheelchair, whereas the controller interlinks the motion platform and the display unit. The roller system in Figure 2 on the motion platform enables both rotational motion of the wheels and the mapping of the wheels' motion into the virtual world. This is facilitated by the force exerted on the rollers as a result of the wheelchair's and the user's weight. The pulse generating rotary encoders mounted on the rollers enable determination of position, velocity, and acceleration of the wheelchair in the virtual space and facilitate the measuring of differential drive motion as the driving wheels in direct contact with the rollers rotate. This generates forward or backward translations in the virtual world with equal angular velocities *ω*_*R*_ and *ω*_*L*_ for the right and left rear wheels, respectively, and rotational translations with *ω*_*R*_ ≠ *ω*_*L*_.

In Figure 2, the actuated force feedback roller (*rx*) rotates at a velocity of ±*ω*. *F*_*x*_ represents the frictional force between one single roller and the wheel, while the variables *ω*_*m*_ and *τ*_*m*_ represent the actuator's angular velocity and transferred torque, respectively. Slip dynamics at the point of contact between the driving wheels and the rollers could be the major source of simulator error that may contribute to inaccurate representation of the wheelchair's dynamics in the simulator. According to [72], slip is defined as the difference between the rotational velocity of the wheels and the actual or absolute velocity of the wheelchair. However, since the actual linear velocity of the wheelchair on the motion platform is zero, the theoretical difference between the rotational velocity of the driving wheel and the rollers is used to account for the possible wheel slip errors in the simulator. A comparison between the wheel's velocity and the motor's current in relation to the torque *τ*_*w*_ of the driving wheel is also made to determine the wheelchair's instability. This involves examining the basic properties of the dc motor required in the theoretical determination of the output torque *τ*_*m*_. Instability is considered if *τ*_*m*_ ≠ *τ*_*w*_. In this case, a method for automobile traction control [73] is used in the stabilisation. However, to avoid the tipping-over instability of the wheelchair on the simulator, fastening straps have been used to hold the wheelchair in position.

Regarding the data collection experiments, an electrical wheelchair is used, with the original embedded joystick as the main interface. To effectively evaluate the steering behaviours of the participants in relation to the general environment, the virtual worlds attempted as much as possible to represent the areas encountered frequently by the participants. Seven individuals participated in the data collection. A few of the data collection experiments are elaborated in Sections 3.3–3.5. The seven participants include a female and six males, aged between 26 and 74. Although none of the participants had a degenerative disability condition or tremors, three were regular wheelchair users while the rest had not used the wheelchair before. The first time users had to familiarise themselves with the steering of wheelchair in both virtual and real environments before the capturing of their steering data could start.  Table 2 presents the participants' information.

**Figure 4:**
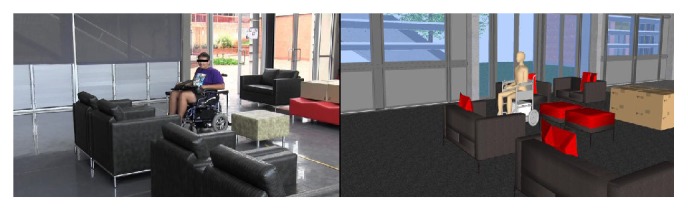
A user steering the wheelchair in a living room setup in both virtual and reality environments.

Five desirable characteristics of the VS-1 platform regarding this study are noted: (1) it guarantees safety of the participant; (2) it eliminates the need for sensor installation; (3) it enables the user to feel the synchronised pitch and roll rotational driving motions on flat and inclined surfaces, reducing the possibility of simulator sickness; (4) experiments can be performed using real manual or electrical wheelchairs; the electrical wheelchair can be steered using the standard embedded joystick or any other available user interface; and finally (5) the virtual world (examples in Figures 4 and 8) provides the user with a close representation of a real environment and a feeling of collision sound.

**Figure 5:**
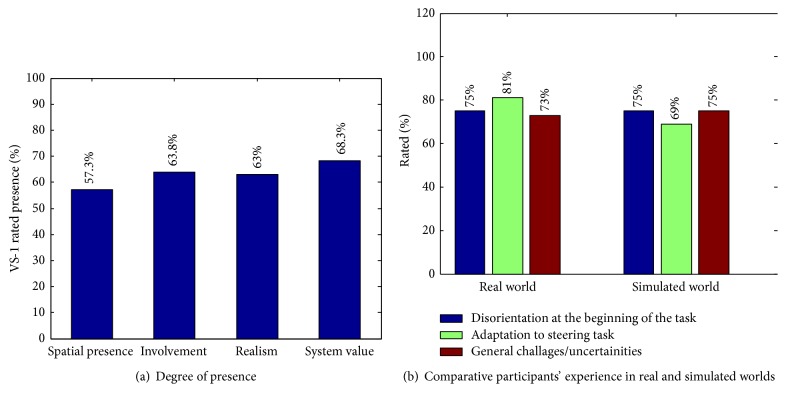
Virtual-Reality System 1 (VS-1): the augmented virtual and motion platform at FSATI for wheelchair simulations.

Notwithstanding the above advantages, the potential usefulness of the motion platform in user evaluation can only be acceptable if the virtual world and the impression of motion in the simulated environment conform to the real world to a certain extent. A study evaluating participants' perception of degree of presence and comparing the usability of the simulated world of VS-1 with the reality world is conducted in [71]. The degree of presence compared to the real world is evaluated in terms of spatial presence, involvement, realism, and system value; a portion of evaluation outcome is presented in Figure 5. Spatial presence indicates the extent to which participants acknowledge their existence in the environment in the actual sense, while involvement concerns system response to user inputs and the resulting motion feedback. Realism is expressed by the use of a real wheelchair and the rotational motion of VS-1 platform, while system value represents the degree to which users recognise the motion platform in general as an evaluation aid. According to the study, the participants experienced 75% disorientation with regard to steering tasks and platform usage at the beginning of evaluation in both reality and simulated world. However, adaptation was much faster in both cases, with 81% adaptation rate in the reality world and 69% in the virtual world. Considering the presented tasks, the participants observed 73% and 75% challenges/uncertainties in the reality and simulated worlds, respectively. The study, thus, demonstrates a fair similarity between the steering experience observed within the virtual world and within the reality world.  Figure 4 for instance, shows the user captured while steering the wheelchair in a living room environment in both virtual and reality worlds during the evaluation.

As in most simulators, the existence of cue conflicts between the motion platform and the virtual world due to lack of the platform's linear motion and the sensory simulation artefacts (such as reduced field of view in the virtual world) must be acknowledged. Moreover in the minds of participants, however important it is, a simulation task will always be perceived as a simulation exercise, with few risks for careless actions and few rewards for desired behaviours. Nevertheless, studies have demonstrated the feasibility of simulation techniques and have shown that simulation results approximate those obtained by other methods [74]. Authors therefore trust the relative validity of VS-1 as sufficient for driver behaviour assessment.

### 3.2. Experimental Data Captured for Behaviour Modelling

While it is apparent that complete success in modelling driver behaviour requires vast information that may not be fully captured by experiments alone, the platform provides the following sensor information for utilisation.

(1) Range or distance between the wheelchair and other objects (*ρ*_obst_).
(2) Range rate or velocity of the wheelchair relative to other objects (*ν*_obst_).
(3) Direction of an object from the position of wheelchair (*ϕ*_obst_).
(4) Absolute velocity of the wheelchair (*ν*_*k*_).
(5) Yaw angle of the wheelchair (*ϕ*_*k*_).

Besides, VS-1 also avails yaw rate, pitch angle, and roll angle.

### 3.3. Experiment 1

**Figure 6:**
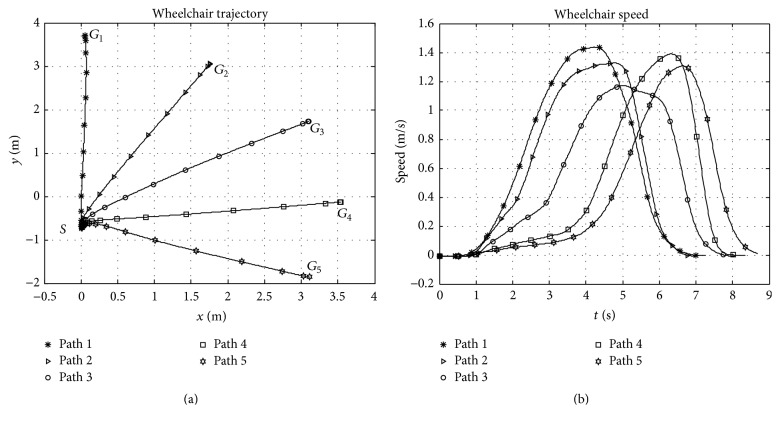
Wheelchair trajectories and speeds observed during experiment 1.

Experiment 1 is conducted in a “risk” free environment. The word “risk” is used in this study to represent the objects or agents that the driver would not wish to steer over or closer to or collide onto. Goal positions *G*_1_ to *G*_5_ are set 4 m away from the starting point *S* at angles 90°, 60°, 30°, 0°, and −30°, respectively. In each trip, the participant is directed to drive five times from position *S* (with wheelchair initially oriented towards *G*_1_) to all the goals. Trajectories and speeds observed during the experiment are shown in Figure 6. It is noticeable that steering towards *G*_1_ involved a steeper rise in steering speed as compared to the rest. More skewed directions of the goal from the initial wheelchair orientation at position *S* result in slower initial accelerations. Explanation regarding this behaviour is considered common knowledge; that drivers constantly perceive an instantaneous or look-ahead goal whose position from the vehicle is a function of the available steering space and path curvature. According to Figure 6, highly skewed global goals involve highly curved paths at the beginning of the journey, implying closer initial instantaneous goals and slower initial accelerations. Desired steering velocity is therefore related to position of the instantaneous goal. Besides, participants prefer aligning themselves to the global goal (if possible) at the initial phases of the journey. Once aligned, the position of the instantaneous goal rapidly shifts towards the global goal and steeper rise in speeds is realised. This is the observable pattern; however the amount of shift with regard to the environmental situation is subjective. It may be concluded, therefore, that the local driving velocity in a risk free environment is related directly to the position of the instantaneous goal and is influenced majorly by the curvature of the path.

### 3.4. Experiment 2

**Figure 7:**
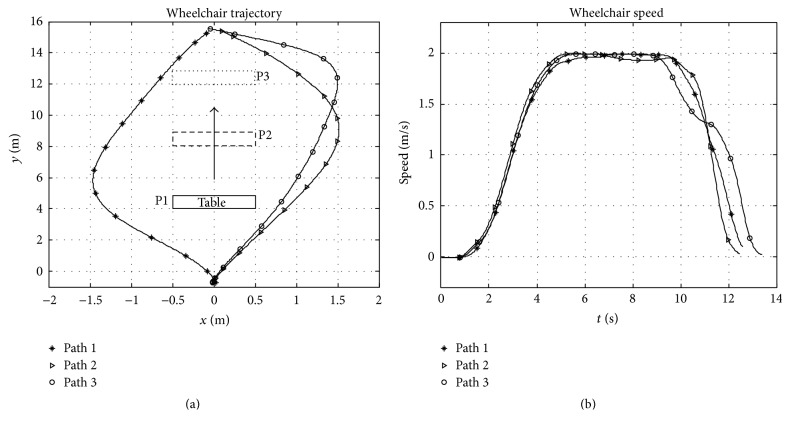
Trajectories and speeds of wheelchair observed during experiment 2.

The second experiment is conducted in a configuration with an object placed 4 m, 8 m, and 12 m away from the starting point in the first, second, and third trip, respectively. In each trip, the participant is advised to drive from the starting point, close to (0,0) in Figure 7, to the goal approximately 15 m away. Figure 7 depicts the trajectories and the speeds captured from one participant. It is observable that although the participants deviate away from the observed risk, the availability of sufficient space within the configuration enables them to choose the paths with little effect on the desired steering speed.

### 3.5. Experiment 3

**Figure 8:**
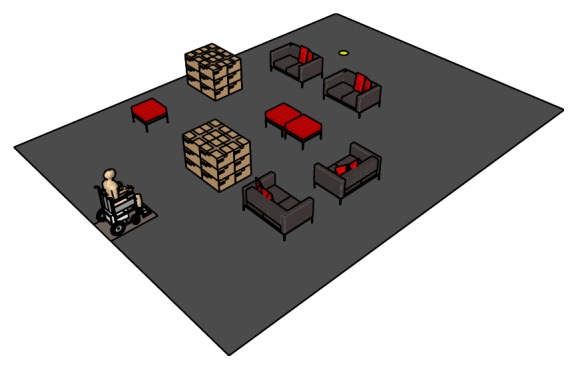
The virtual living room environment considered for experiment for experiment 3, perimeter wall not shown for clarity reason.

**Figure 9:**
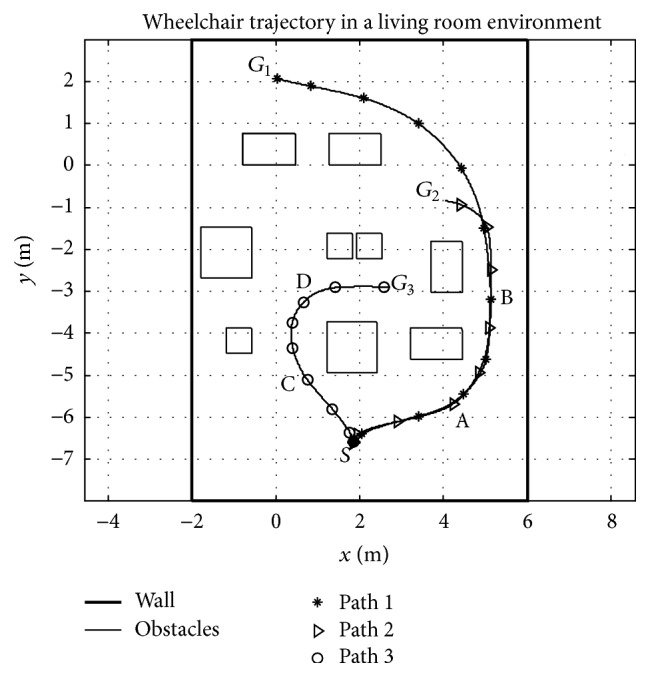
Wheelchair trajectories observed during experiment 3. The rectangular shapes in the configuration space represent the living room furniture.

**Figure 10:**
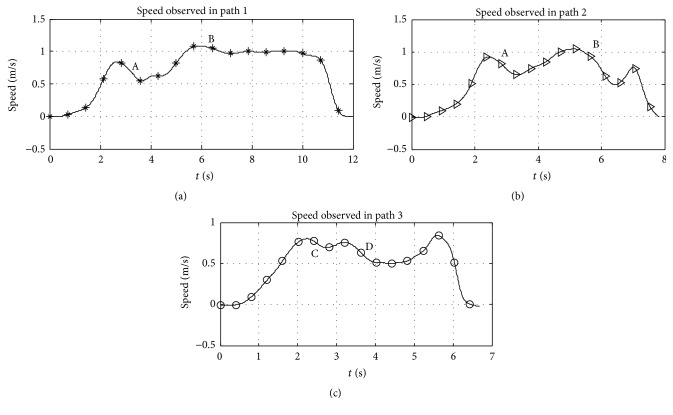
Wheelchair speeds observed during experiment 3 in the living room experiment.

The third experiment observes the steering behaviour of the participants in the living room environment depicted in Figures 4 and 8. In this experiment, participants are advised to drive five times to the goals *G*_1_, *G*_2_, and *G*_3_ from the starting point *S*, without speed or path restrictions. Interestingly most participants considered the paths and speeds depicted in Figure 9, allocating additional local priority to the local risk compared to the global goal. It may be important, also, to note that participants preferred longer safer paths as opposed to shorter risky paths; the “magnitude of risk” in this case is determined by the amount steering accuracy required to avoid collision. At positions A, B, C, and D, the apparent possibility of collision with furniture and reduction in the immediate forward space along the perceived curved path compels participants to observe closer instantaneous goals; this accordingly resulted in the reduction of the steering speed at the respective points as depicted in Figure 10.

## 4. Driver Behaviour Modelling

According to the* intentional stance* strategy, Dennett [75] treats an entity (an organism or artefact) as a rational agent having the ability to regulate its choice of action by its desires and beliefs. Dennett then defines “behaviour” as a goal oriented activity of an agent that can only be understood by assigning intentions or goals to the agent. Modelling the driving behaviour of an individual, thus, involves defining one of the numerous goals that a driver may be required to reach. Generally, different drivers demonstrate different steering actions and reactions within the same environment to achieve the same objective. These subjective behaviours are commonly related to the driver's capability in terms of decision making (choice) and risk taking (desires) and are affected by personality, experience, state of driver, task demand, and environment. Adapting an artefact to exhibit the desired characteristics of an individual and to take into account the evolving and dynamic behaviour of users may thus be approached in two ways, namely:

1. *System Training.* Here, the model learns by observing over time the way human drivers execute particular tasks. Once a task is perfectly learned, the model can proceed to learn other tasks. This approach could be applicable to motor-vehicle driving tasks because of the regular nature of the motor-vehicle driving and the fact that most vehicle-driving tasks are well defined and easily representable by heuristics. In the wheelchair, however, the workspace is very complex and undefined, with various tasks lacking the chronological rules of execution.
2. *Representing the Entire Driving Behaviour Theoretically*. This approach has been considered in this paper. All local tasks performed by the driver are considered together to realise the driving behaviour. Theoretical driver behaviour models of this nature are initially limited in scope and may not perfectly represent the behaviours considered specific or specialised in nature. However, they can be advanced over time to closely predict the actual driving behaviour. These theoretical driving models may be validated by comparing their outputs against some real data from human drivers.

### 4.1. Dynamic Representation of Driving Behaviour

Four major factors are, therefore, considered to influence wheelchair driving as follows. The first prompts the user to exert some force to begin or continue in motion pertains to the difference between the actual wheelchair position and the target position (in this case the instantaneous goal). This factor is the primary motivational element that instigates the driver to move; as long as it exists, the wheelchair driver is understood to apply and continue applying the driving force. The second factor influences the amount of force exerted in “attempt” to minimise the positional difference. This factor, the desired velocity, is related to the urgency or average time required by the user to accomplish the driving task at hand; and it is usually a function of disposition and the prevailing personal desire and priority of the driver. The third factor concerns risk assessment and involves both the driving capability of the user and the driver's safety opinion of the environment. All these factors contribute concurrently leading to variations in wheelchair velocity while in motion towards the goal. Besides, there exists an interrelationship between the three factors in that the users establish a subjective constant risk level and when this is exceeded, a compensation mechanism is activated. For instance, this may involve altering the position of the instantaneous goal, which then alters the direction and speed of driving. Finally, it is important to observe that the amount of force exerted is constrained by physical limits of the wheelchair. The local driving velocity *ν*, limited by maximum wheelchair velocity *ν*_max_, is therefore considered to be a function of goal *ν*_des_(*g*_*i*_) and environmental situation *ν*_env_(env) as presented in

(8) $$\begin{array}{c} \nu =\left|\;\nu_{des}\left(\;g_{i}\;\right)+\nu_{env}\left(\;env\;\right)\;\right|\leq \nu_{max}. \end{array}$$

### 4.2. Desired Steering Velocity

Drivers are generally believed to prefer some constant driving speeds in environments with minimal risk factors. Desired speed is a personality factor that varies from one individual to the other. It is affected not only by composition of the workspace but also by the implied steering complexity and user experience. The composition of the workspace introduces an aspect of risk and safety which compels a driver to take on some adaptation mechanisms in order to limit the perceived risk to an acceptable subjective threshold. Such mechanisms generally confine the local driving speed to a safe minimum. A discussion in this regard is presented in Section 4.3. The steering complexity on the other hand pertains to influence of complex orientational manoeuvres involved in the steering task, including effects path curvature. Disassociating the influence of risks resulting from environmental configuration, from the desired speed, and considering desired speed as a function of the steering complexity alone result in (9), where the desired velocity is considered to be a function of path curvature in the direction of the instantaneous goal. As presented in (9), desired velocity only takes the observable variables having a systematic relationship with the steering behaviour into consideration and avoids the effects of nonquantifiable subjective factors including user experience and task urgencies:

(9) $$\begin{array}{c} \nu_{des}=s_{des}\cos^{p}\left(\;\phi_{k}-\phi_{k-1}\;\right)e, \end{array}$$

where *p* is a constant, *s*_des_ is the desired driving speed, *ϕ*_*k*_ is the direction of the wheelchair at time instant *k*, and cos^*p*^⁡(*ϕ*_*k*_ − *ϕ*_*k*−1_) is the influence of path curvature on desired velocity. **e** as expressed by (10) is the direction of desired velocity:

(10) $$\begin{array}{c} e=\frac{q_{L_{k}}-q_{k}}{\left|q_{L_{k}}-q_{k}\right|}. \end{array}$$

In (10), **q**_*L*_*k*__ is the position of the instantaneous goal, while **q**_*k*_ is the instantaneous position of the wheelchair.

### 4.3. Influence of Risk and User Adaptation Mechanism

Collision or threat avoidance and goal-seeking reactions constitute the driver's fundamental behaviours. In fact, in most cases, the capability of a wheelchair driver is evaluated based on the ability to avoid threats and collisions during steering. Besides, common wheelchair accidents that have resulted in severe wheelchair damages and injuries to the driver can be related to collision. Collision avoidance is, therefore, elemental to the safety of both the wheelchair and the user. Drivers generally presume some constant risk thresholds and safety margins that they seek to observe in the vicinity of danger during steering. When such thresholds are exceeded, certain risk-compensating mechanisms are initiated to minimise the risk level. In the Taylor's risk-speed compensation model [76], it is observed that drivers regulate their driving speeds in accordance with the magnitude of the perceived risk in such a way that larger magnitudes result in slower speeds. In order to adapt the wheelchair to such behaviours and eliminate the common variations in the drivers' level of attention, proper risk detection systems need to be instituted on the wheelchair. The following two hypotheses are proposed in this paper as the main adaptation references commonly presumed by the drivers to confine wheelchair within the limits of safety:

1. Time-to-risk (TTR).
2. Distance-to-risk (DTR).

**Figure 11:**
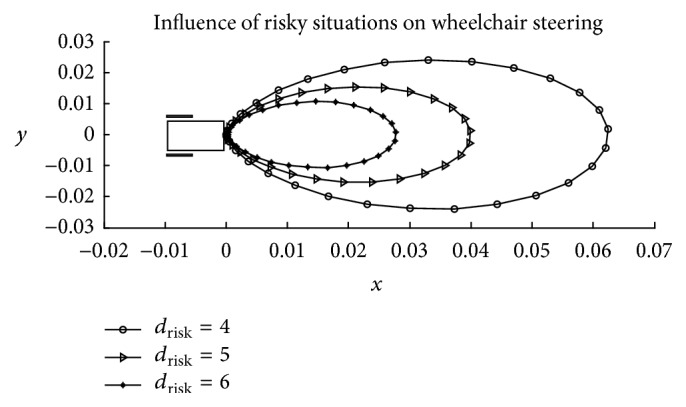
Influence of risky situations on wheelchair steering with *m* and *n* = 2 and with distance-to-risk *d*_risk_ considered as the main user adaptation reference.

The time-to-contact with a risk is a function of both the wheelchair's distance from the risk and speed of travel towards the risk. The consideration of TTR naturally implies that the wheelchair can reach or get very close to risky objects at very low velocities. On the other hand, DTR means that the driver will maintain a comfortable distance from the risk. Expression (11) is considered to represent the drivers' avoidance behaviour in the vicinity of risks:

(11) $$\begin{array}{c} \nu_{env_{k}}=-k_{env}\overset{N}{\underset{i=1}{\sum }}\frac{\cos^{m}\left(\phi_{obst_{i}}-\phi_{k}\right)}{A_{i}^{n}}, \end{array}$$

where *k*_env_, *m*, *n*, and *N* are constants, *ϕ*_obst_*i*__ is the instantaneous direction of point *i* of the risk from the position of wheelchair, *ϕ*_*k*_ is wheelchair direction at time instant *k*, and *A* is the adaptation mechanism presumed by the user.  Figure 11 depicts the variation of (11) with respect to different positions and directions of risks in the workspace, with DTR presumed as the main adaptation reference. The strength of (11) is based on the aspect that only risks within the field of view affect steering behaviour. Risks considered closer and directed to the viewer have greater influence compared to those viewed as skewed and further away. It, therefore, scales down the workspace to a smaller workable field of consideration. Taking both (9) and (11) into account, the model considered to represent the driver's behaviour in the local context is represented by

(12) $$\begin{array}{c} \nu_{k+1}=\nu_{k}+k_{\nu }\left(\;\nu_{des}-\nu_{k}\;\right)-k_{env}\overset{N}{\underset{i=1}{\sum }}\frac{\cos^{m}\left(\phi_{obst_{i}}-\phi_{k}\right)}{A_{i}^{n}}. \end{array}$$

## 5. Simulation, Results, and Discussion

### 5.1. Parameter Identification and Adaptation Mechanism

The linearity in parameters of the proposed driver behaviour model enabled the consideration of ordinary least squares method in the identification of parameters. In addition, the moving average filter with a span of 20 is used in smoothing the captured data. The result of regression analysis of the model is presented first in Table 3, where the DTR criteria presented in (13) are considered as the primary adaptation mechanism adopted by the participants to avoid collision risks, and also in Table 4, where TTR in (14) is the primary adaptation mechanism:

(13) DTRk=ρobstkiq^i+ρobstkjq^j,

(14) TTRk=ρobstkiνkiq^i+ρobstkjνkjq^j.

In (13) and (14), *ρ*_obst_*k*__ = **q**_obst_ − **q**_*k*_ is the instantaneous distance between the risk at position **q**_obst_ and the wheelchair at position **q**_*k*_, while *ν*_*k*_ is the instantaneous velocity of the wheelchair.

Table 3 contains the model constant *p*, the identified parameter values, standard error in value and percentage, *t*-statistics, maximum deviation between the fitted and the observed data, and coefficients of determination of the analysis for each of the seven participants. The optimised values of constants *m* and *n* used in the identification process are 4 and 2, respectively. These values represent a pair that resulted in the highest coefficient of determination with regards to most participants. In Figure 11, the value of constant *m* defines the shape of the contours along which risks possess the same magnitude of influence; a higher value indicates that the driver is less bothered about skewed risks as compared to risks perceived along or closer to the direction of the wheelchair. Referring to (11), *m* = 1 results in a circular contour, while higher values (*m* > 1) produce oval contour shapes. The high value of constant *m* considered in the identification process thus represents the reduced influence of side risks on the participant's steering behaviour. Constant *n* on the other hand determines the magnitude of risk influence based on the presumed adaptation mechanism. Higher values imply that the magnitude of influence of the observed risk is considerably high within the close neighbourhood but negligible outside the neighbourhood.

In parameter identification, navigation data exceeding 80,000 data sets per participant, obtained from the previous steering experiments, were collected and utilised. Out of the captured data, 85% are used in parameter identification, to ensure that the observed parameters represent well the natural behaviour of the participant, while only 15% are used in curve fitting validation. The observed values of *R* ^2^ in Table 3 demonstrate how well the model replicates the collected data. Besides, the resulting large absolute values of *t*-statistics established the significance of the identified coefficients in the behaviour model. It is noticeable that higher values of *t*-statistics corresponding to *k*_*ν*_ as compared to *k*_env_ are obtained, which indicates the stronger impact of desired velocity as compared to risks avoidance, which could be influenced by the drivers' subjective goal reaching urgencies during the steering experiments. The variability of *ν*_des_ from one participant to the other also demonstrates the importance of identifying the individual driver's behaviour, because different drivers prefer particular driving speeds.

Similar results are presented in Table 4. The same constants in Table 3 are also considered in comparing the relevance of the two hypothesised risk adaptation mechanisms. TTR is adopted and the observed results are found to be very close to those in Table 3. Nonetheless, one can quickly realise the slightly lower coefficient of determination and *t*-statistics obtained with the consideration of TTR. Besides, the parameters obtained with respect to participants 6 and 7 may not represent the actual behaviour, because *ν*_des_ and *k*_*ν*_ are negative. Moreover, the desired velocity obtained for participant 7 seems unreasonable. These may have resulted from the drivers' preference and their choice of adaptation criteria. Because of the slow wheelchair speed, driver with confidence on the braking system may for instance only observe the distance to the risk and apply an instant brake at sufficient distance just before collision. It may be considered that TTR is not observed if there is no progressive reduction in the speed as the driver approaches the threat, the use of TTR in this case may produce the observed invalid results. It may therefore be concluded that the consideration of DTR as the principal adaptation criteria that wheelchair drivers adopt in the vicinity of risks corresponds well with most wheelchair drivers.

### 5.2. Trajectory Fitting

**Figure 12:**
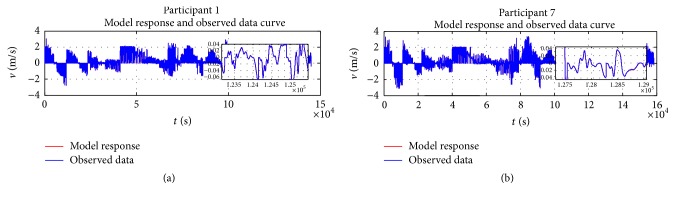
Captured linear velocity and model response for participants 1 and 7.

**Figure 13:**
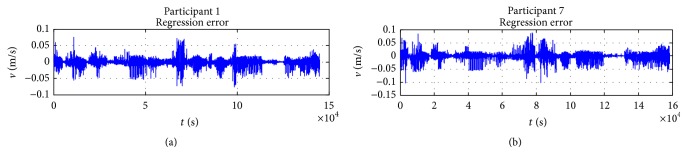
Captured linear velocity and model response for participants 1 and 7.

**Figure 14:**
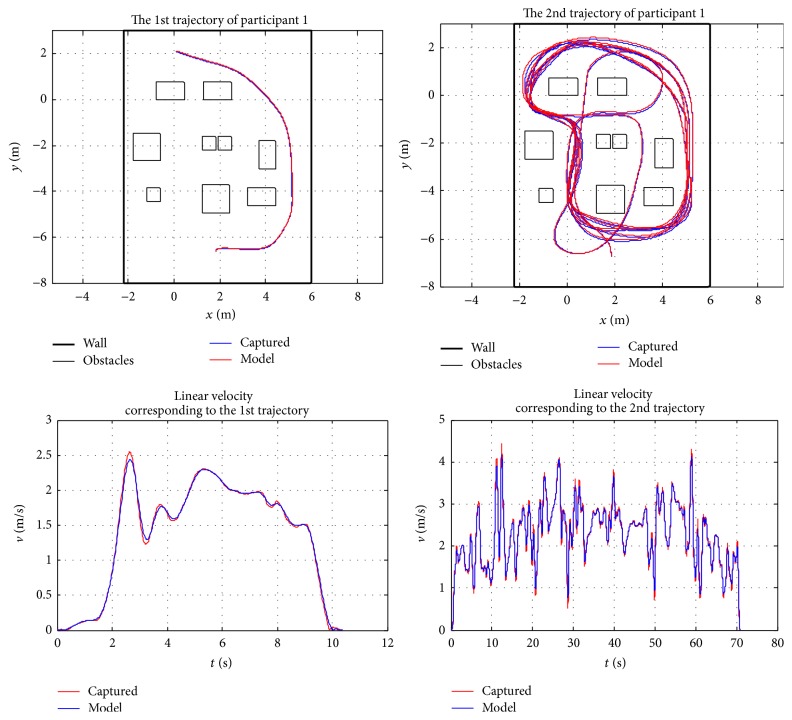
Observed trajectories and linear velocities of participant 1.

**Figure 15:**
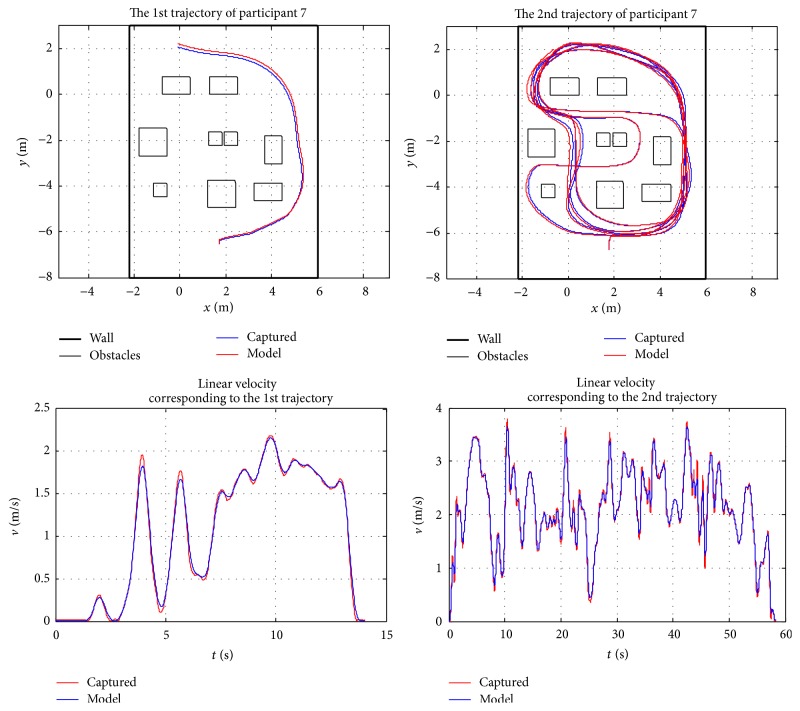
Observed trajectories and linear velocities of participant 7.

Because of the space limitation, only two randomly chosen participants' results are presented in this section to validate the behaviour model. The presented curves include a comparison between the model and captured data, observed error between the model and captured data, and wheelchair trajectory and steering velocity for the two participants.  Figure 12 depicts the relationship between the observed data and model response; it shows the large amount of data used in the least squares estimation of the model parameters presented in Table 3 for each participant. With these constants and parameters, it is interesting to observe how the model closely represents captured data. The observed difference between the model and captured data as presented in Figure 13 basically represents white noise. The observed and generated trajectories and linear velocities of participant 1 and participant 7 are depicted in Figures 14 and 15, respectively. The depicted comparison of the “model generated” trajectories and linear velocities with the corresponding trajectories and linear velocities observed from real data in Figures 14 and 15 demonstrate the good correspondence between the model and the actual behaviour.

### 5.3. A Comparison with Emam et al. [18]'s Driver Behaviour Model

**Figure 16:**
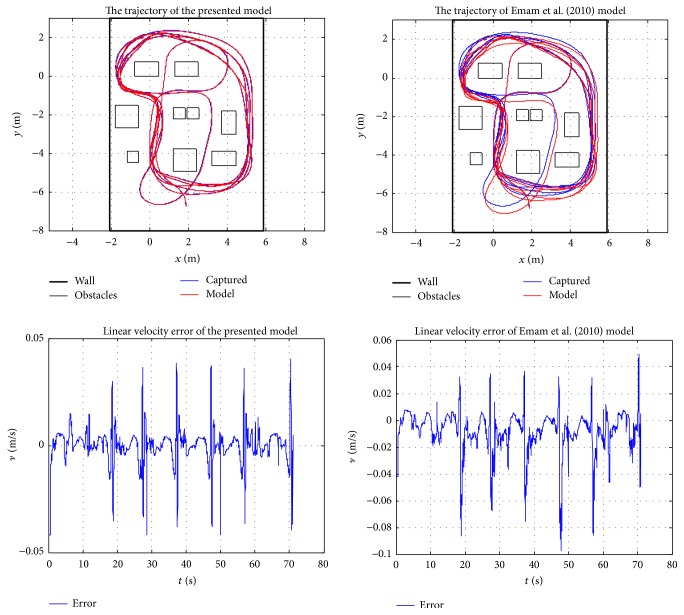
The curve fitting comparison between the presented model and Emam et al. [18]'s model.

A curve fitting comparison between the presented model and Emam et al.'s model [18] is also presented in Figure 16. The second trajectory of participant 1 (presented in Figure 14) is used in the comparison, with DTR as the adaptation criteria. It is noticeable that the presented model performs better, with a very close fitting compared to Emam et al.'s model. In addition, Table 5 also presents the estimated parameter values, standard error, *t*-statistics, maximum deviation, and *r*-squared for Emam et al.'s model with respect to the seven participants. Comparing the regression analysis in Table 3 with the analysis in Table 5, it is apparent with comparatively higher standard errors and maximum deviation that the presented linear model still performs better. Besides, some negative parameters value were obtained during the identification.

## 6. Conclusion

The primary objective of this study was to develop a model that represents the local steering behaviour of a wheelchair driver. This paper has presented a good driver behaviour model that is also linear in parameters. The model assumes explicit knowledge of subsequent intentions of the driver, in order to generate the adaptation signals that may be required to adapt the wheelchair to the driver's steering behaviour. It is observable from the identified parameters that although participants exhibited similar driving behaviours, there is always an implied uniqueness with each participant, which validates the need for modelling and identification of the driver's behaviour. This is more important, especially, for the ageing users whose steering capabilities deteriorate with time. Due to simplicity and linearity of the model, the ordinary least square method has been used in the determination of model parameters. The regression analysis of the model with identified parameter values have demonstrated acceptable performance results for all participants.

## 7. Recommendation for Future Works

In this study, explicit driver intention is assumed to be known. However not all wheelchair users have the capability of communicating properly all the navigational commands required to make the wheelchair move, stop, or turn with the available user interface. Complete assistance demands a model that can utilise the available information to predict the driver's intention, to assist a disadvantaged driver to steer in the right direction. Incorporating an intention detection model in the driver behaviour model may therefore be considered if the model is to be used as a codriver to provide real-time assistance to the driver.

## Figures and Tables

**Table 1:** Comparison of the potential field modifications based on their applicability in the formulation steering behaviour of wheelchair users.

	APF	EAPF	PEAPF	BPF	DPF
Smooth planning	✓	✓	✓	✓	✓
Local planning	✓	✓	✓	✓	✓
Global planning		✓	✓	✓	
Complex environments			✓	✓	✓
Highly scalable			✓	✓	✓
Directionality					✓
Min. computational time					✓

**Table 2:** Information about the seven participants who took part in the steering task for data collection.

	Age	Sex	Usage
Participant 1	50	Male	Never
Participant 2	26	Male	Never
Participant 3	52	Male	Regular
Participant 4	60	Male	Never
Participant 5	74	Male	Regular
Participant 6	30	Female	Regular
Participant 7	46	Male	Never

**Table 3:** Statistical analysis of the model with DTR being the adaptation mechanism. Indicated constants represent a pair that resulted in the highest coefficient of determination.

*p*	Parameters		Std. error		1/*t*_stat_	Max. Dev.	*R* ^2^
Participant 1							
2	*k*_*ν*_	0.0023296	0.0000493	2.12%	0.021145	0.0066778	0.9998727
	*k*_env_	0.0000607	0.0000042	6.92%	0.069456		
	*ν*_des_	2.1620485	0.0000664	0.003%	0.000031		

Participant 2							
1	*k*_*ν*_	0.0013058	0.0000457	3.50%	0.034998	0.0044821	0.9998751
	*k*_env_	0.0000168	0.0000050	29.8%	0.297619		
	*ν*_des_	3.1936471	0.0000572	0.002%	1.791*e* − 5		

Participant 3							
2	*k*_*ν*_	0.0016111	0.0000638	3.96%	0.039600	0.0282700	0.9998215
	*k*_env_	0.0001812	0.0000042	2.32%	0.023179		
	*ν*_des_	2.9748170	0.0000809	0.003%	2.720*e* − 5		

Participant 4							
1	*k*_*ν*_	0.0022988	0.0000481	2.10%	0.020924	0.0096421	0.9998336
	*k*_env_	0.0001566	0.0000041	2.62%	0.026181		
	*ν*_des_	1.5590495	0.0000483	0.003%	3.098*e* − 5		

Participant 5							
1	*k*_*ν*_	0.0011705	0.0000330	2.82%	0.028193	0.0048858	0.9999171
	*k*_env_	0.0000525	0.0000024	4.57%	0.045714		
	*ν*_des_	2.3263703	0.0000322	0.001%	1.384*e* − 5		

Participant 6							
2	*k*_*ν*_	0.0033319	0.0003469	10.4%	0.104115	0.0288915	0.9992932
	*k*_env_	0.0002114	0.0000097	4.59%	0.045885		
	*ν*_des_	2.0650043	0.0003439	0.017%	1.665*e* − 4		

Participant 7							
2	*k*_*ν*_	0.0007837	0.0000496	6.33%	0.063290	0.0108345	0.9998355
	*k*_env_	0.0001583	0.0000048	3.03%	0.030322		
	*ν*_des_	5.5191529	0.0000688	0.001%	1.247*e* − 5		

**Table 4:** Statistical analysis of the model with TTR being the adaptation mechanism and for the same constants as those in Table 3.

*p*	Parameters		Std. error		1/*t*_stat_	Max. Dev.	*R* ^2^
Participant 1							
2	*k*_*ν*_	0.0022305	0.0000508	2.28%	0.022775	0.0051076	0.9998726
	*k*_env_	0.0000116	0.0000024	20.7%	0.206897		
	*ν*_des_	2.1419324	0.0000642	0.003%	2.997*e* − 5		

Participant 2							
1	*k*_*ν*_	0.0012839	0.0000472	3.68%	0.036763	0.0042400	0.9998751
	*k*_env_	0.0000059	0.0000018	30.51%	0.305085		
	*ν*_des_	3.2207044	0.0000570	0.002%	1.770*e* − 5		

Participant 3							
2	*k*_*ν*_	0.0011325	0.0000646	5.70%	0.057042	0.0077142	0.9998197
	*k*_env_	0.0000370	0.0000027	7.30%	0.072973		
	*ν*_des_	3.3136044	0.0000776	0.002%	2.342*e* − 5		

Participant 4							
1	*k*_*ν*_	0.0017910	0.0000473	2.64%	0.026410	0.0053594	0.9998327
	*k*_env_	0.0000718	0.0000043	5.99%	0.059889		
	*ν*_des_	1.5635212	0.0000435	0.003%	2.782*e* − 5		

Participant 5							
1	*k*_*ν*_	0.0010542	0.0000339	3.22%	0.032157	0.0042527	0.9999171
	*k*_env_	0.0000403	0.0000018	4.47%	0.044665		
	*ν*_des_	2.4670341	0.0000319	0.001%	1.293*e* − 05		

Participant 6							
2	*k*_*ν*_	−0.001482	0.0004203	−28.4%	−0.28360	0.0274354	0.9992838
	*k*_env_	0.0002261	0.0000146	6.46%	0.064573		
	*ν*_des_	−1.513273	0.0002957	−0.02%	−1.95*e* − 4		

Participant 7							
2	*k*_*ν*_	−0.000199	0.0000442	−22.2%	−0.22211	0.0059510	0.9998381
	*k*_env_	0.0000659	0.0000031	4.70%	0.047041		
	*ν*_des_	−13.54669	0.0000522	−0.00%	−3.85*e* − 6		

**Table 5:** The regression parameters obtained with Emam et al.'s model.

	Param. value	Std. error		1/*t*_stat_	Max. Dev.	*R* ^2^
Participant 1						
*K*(1)	−0.00097	0.00033	−34.184%	−0.3418	0.39777	0.99258
*K*(2)	−0.99680	0.00074	−0.0744%	−7.44*e* − 4		
*K*(3)	0.002372	0.00132	55.4434%	0.55443		
*K*(4)	2.666837	0.47865	17.9482%	0.17948		

Participant 2						
*K*(1)	0.000822	0.00169	2.062*e*2%	2.06227	0.55602	0.99266
*K*(2)	−0.99650	0.00075	−0.0756%	−7.56*e* − 4		
*K*(3)	0.002030	0.00117	57.8637%	0.57864		
*K*(4)	2.914947	0.51880	17.7981%	0.17798		

Participant 3						
*K*(1)	0.007529	0.00099	13.1842%	0.13184	0.36037	0.99245
*K*(2)	−0.99562	0.00063	−0.0629%	−6.29*e* − 4		
*K*(3)	0.001935	0.00106	54.7612%	0.54761		
*K*(4)	2.720196	0.51956	19.1000%	0.19100		

Participant 4						
*K*(1)	5.4794*e* − 5	2.577*e* − 5	47.0383%	0.47038	0.58585	0.99193
*K*(2)	−0.99611	0.00053	−0.0528%	−5.28*e* − 4		
*K*(3)	0.000754	0.00028	37.4431%	0.37443		
*K*(4)	3.414773	0.31000	9.07842%	0.09078		

Participant 5						
*K*(1)	0.001279	0.00086	67.1672%	0.67167	0.29464	0.99237
*K*(2)	−0.99631	0.00069	−0.0693%	−6.93*e* − 4		
*K*(3)	0.001302	0.00093	71.2194%	0.71219		
*K*(4)	2.802759	0.61846	22.0662%	0.22066		

Participant 6						
*K*(1)	0.003097	0.00096	30.9182%	0.30918	0.01506	0.99205
*K*(2)	−0.99582	0.00063	−0.0633%	−6.33*e* − 4		
*K*(3)	−0.00016	0.00123	−7.82*e*2%	−7.8198		
*K*(4)	0.645253	11.9476	1.851*e*3%	18.5161		

Participant 7						
*K*(1)	0.002894	0.00192	66.2751%	0.66275	0.66898	0.99254
*K*(2)	−0.99636	0.00071	−0.0708%	−7.08*e* − 4		
*K*(3)	0.001286	0.00064	49.5301%	2.01897		
*K*(4)	3.589096	0.46016	12.8211%	0.12821		
